# Functional Characterization of the N-Terminal C2 Domain from* Arabidopsis thaliana* Phospholipase D*α* and D*β*

**DOI:** 10.1155/2016/2721719

**Published:** 2016-12-22

**Authors:** Renaud Rahier, Alexandre Noiriel, Abdelkarim Abousalham

**Affiliations:** Institut de Chimie et de Biochimie Moléculaires et Supramoléculaires (ICBMS), Univ Lyon, Université Lyon 1, UMR 5246 CNRS, Métabolisme, Enzymes et Mécanismes Moléculaires (MEM), F-69622 Villeurbanne cedex, France

## Abstract

Most of plant phospholipases D (PLD) exhibit a C2-lipid binding domain of around 130 amino acid residues at their N-terminal region, involved in their Ca^2+^-dependent membrane binding. In this study, we expressed and partially purified catalytically active PLD*α* from* Arabidopsis thaliana* (AtPLD*α*) in the yeast* Pichia pastoris*. The N-terminal amino acid sequence of the recombinant AtPLD*α* was found to be NVEETIGV and thus to lack the first 35 amino acid belonging to the C2 domain, as found in other recombinant or plant purified PLDs. To investigate the impact of such a cleavage on the functionality of C2 domains, we expressed, in* E. coli*, purified, and refolded the mature-like form of the C2 domain of the AtPLD*α* along with its equivalent C2 domain of the AtPLD*β*, for the sake of comparison. Using Förster Resonance Energy Transfer and dot-blot assays, both C2 domains were shown to bind phosphatidylglycerol in a Ca^2+^-independent manner while phosphatidic acid and phosphatidylserine binding were found to be enhanced in the presence of Ca^2+^. Amino acid sequence alignment and molecular modeling of both C2 domains with known C2 domain structures revealed the presence of a novel Ca^2+^-binding site within the C2 domain of AtPLD*α*.

## 1. Introduction

Phospholipase D (PLD, EC 3.1.4.4) is a lipolytic enzyme which has gradually emerged, over recent decades, as one of the key components of a broad range of cellular and physiological processes (see [1] for review). PLD hydrolyzes the distal phosphodiester bond of glycerophospholipids, leading to the formation of phosphatidic acid (PA) and to the release of a soluble head group. In addition to the hydrolysis reaction, PLD is able to catalyze a transphosphatidylation reaction in which a primary alcohol is transferred to the phosphatidyl moiety of the substrate phospholipid, leading eventually to the formation of the corresponding phosphatidyl alcohol [2].

Historically, plants have been used as an outstanding model for PLD research. Indeed, PLD activity has been originally described in carrot [3] and the gene was cloned for the first time from* Ricinus communis *[4]. These studies have been the premise of PLD research, allowing a subsequent identification and characterization of PLD homologues in a wide range of organisms, from both prokaryotic and eukaryotic origins, including mammals, yeasts, bacteria, and viruses (see [1] for review).

So far, 12 putative PLD genes have been identified in the plant genetic model* Arabidopsis thaliana *[5, 6]. All these PLDs have been demonstrated to play central roles in seed germination [7, 8], growth and biomass accumulation [9], stress [10, 11], defense [12], and wound healing [13]. Based on their gene architecture, their sequence similarities, and their biochemical properties, these PLDs can be grouped into two major categories. The C2-PLD category comprises 10 PLD genes that harbor a C2 domain, subdivided into *α*-, *β*-, *γ*-, *δ*-, and *ε*-type PLDs. In contrast, the PX/PH-PLD category presents only 2 PLD genes that contain a phox domain (PX) and a pleckstrin homology (PH) domain, similarly to mammalian PLDs.

The *α*-type PLD comprises 3 isoforms involved in hyperosmotic stresses [14] and senescence presumably by transducing phytohormone signals [15]. In vitro, *α*-type PLD from various plant sources has been shown to hydrolyze phosphatidylcholine (PC), phosphatidylethanolamine (PE), and phosphatidylglycerol (PG) in the presence of 20 to 100 mM of Ca^2+^ and detergents, at physiological pH [16–21]. Nevertheless, using the recombinant* Ricinus communis *PLD*α* (RcPLD*α*), Pappan and Wang [22] also demonstrated that at an acidic pH, *α*-type PLD has a basal hydrolytic activity towards PC vesicles containing both PE and phosphatidylinositol-4,5-bisphosphate (PIP_2_), in the absence of Ca^2+^, and is stimulated by Ca^2+^ concentrations ranging from 10 *μ*M to 500 *μ*M [22]. The *β*-type PLD, composed of 2 isoforms, has been demonstrated to be implicated in the regulation of actin polymerization/depolymerization and was proposed to play an important role for tip growth of pollen tubes [23]. The *γ*-type PLD, composed of 3 isoforms, has been shown to be involved in lipid modulation under aluminum stress [24]. In contrast to the *α*-type PLD, neither *β*- nor *γ*-type PLDs require detergent for their activity and are most active in vitro at micromolar calcium concentrations. Nevertheless, both of these isoforms have been demonstrated to hydrolyze PC, PG, and N-acylphosphatidylethanolamine in the presence of PIP_2_ and 50 *μ*M of Ca^2+^, at a physiological pH [16]. Concerning the *δ*-type PLD, 2 spliced variant isoforms have been identified so far and shown to be involved in defense signaling [12] and freezing tolerance [25]. Similarly to *β*- and *γ*-type PLDs, *δ*-type PLD hydrolyzes PC in the presence of PIP_2_ and 50 to 100 *μ*M of Ca^2+^ but is also stimulated by oleate [26]. Unlike the other members of the* Arabidopsis* PLD superfamily, the PLD*ε* represents the unique member of its class. This PLD, which was originally grouped into the *α*-type subfamily (PLD*α* 4), appears to promote plant growth by acting on nitrogen signaling [9]. Interestingly, among all plant PLDs, the PLD*ε* is the most permissive in terms of enzyme activity conditions requirements. Indeed, this PLD can be assayed in the presence of detergents and Ca^2+^ concentrations in the mM range but also in the presence of oleate, PIP_2_, and *μ*M concentrations of Ca^2+^ [9].

The C2-PLD category presents striking differences in terms of catalytic properties and regulatory processes [5, 6]; all members of this group exhibit a Ca^2+^-dependent hydrolysis of phospholipids. This feature has been attributed to the presence of the Ca^2+^-dependent membrane-targeting C2 domain. This domain is an independent folding module of approximately 130 residues long that has been first described as the second conserved domain of the Ca^2+^-dependent protein kinase C (PKC conserved 2) [27]. Subsequently, an increasing number of C2 domains have been identified in a broad range of membrane-associated proteins, such as Synaptotagmin [28], involved in vesicular trafficking, or the cytosolic phospholipase A_2_ (cPLA_2_) [29, 30] as well as the phospholipase C-*δ* (PLC*δ*) [31], both involved in lipid signaling and metabolism.

All of the C2 domains described so far present a conserved *β*-sandwich fold, composed of eight antiparallel *β* sheets that can be separated into type I and type II topologies, which slightly differ in their *β*-strand connectivity [32]. Indeed, the first and the second *β*-strands (*β*1 and *β*2) of the type I topology correspond, respectively, to the last and the first *β*-strands (*β*8 and *β*1) of the type II topology [32]. Nevertheless, this secondary structure succession is highly conserved and is connected along with three variable loops, which harbor several acidic residues, identified as Ca^2+^ binding regions (CBR). Indeed, the tertiary structure configuration of C2 domains drives to a spatial proximity of CBRs which act as a Ca^2+^ binding pocket [32, 33]. Therefore, C2 domains are able to specifically bind one to three Ca^2+^ ions, leading to a conformational change of the whole enzyme and, eventually, to its recruitment at the membrane.

Indeed, Ca^2+^ has been shown to regulate proteins behavior as demonstrated for cPLA_2_, by slowing down its membrane dissociation constant or, for PKC*α*, by modulating both its membrane association and dissociation rates. Interestingly, Ca^2+^ binding studies on full lengths cPLA_2_ and PKC*α*, in comparison to their respective C2 domains alone, have shown distinct effect of each bound Ca^2+^ ion on proteins behavior. Indeed, site directed mutagenesis of Ca^2+^ coordinating residues, in association with lipid monolayer and surface plasmon resonance studies, has revealed that one Ca^2+^-bound to the C2 of the cPLA_2_ leads to an intradomain conformational change, which is critical for its membrane penetration, while the other bound Ca^2+^ is not directly involved in the membrane binding [34]. In contrast for PKC*α*, authors have shown that one Ca^2+^-bound to the C2 induces an interdomain conformational, implying the whole enzyme and, thus, leading to the membrane binding of non-C2 residues. The other bound Ca^2+^ may be involved in more complex interactions with the membrane, including the binding to phosphatidylserine (PS) [34].

Similarly, C2 domains of plant PLDs have been demonstrated to exhibit several of the conserved acidic residues within their CBRs [32, 33]. However, despite the fact that the recombinant domains are able to bind Ca^2+^, striking differences can be observed concerning their binding affinities. Indeed, as reported by Zheng et al. [35], the C2 domain from* Arabidopsis thaliana* PLD*α* (AtPLD*α*) is able to bind 1 to 3 Ca^2+^ with a low affinity (Kd ranging from 470 to 590 *μ*M) while the C2^Δ173–190^ domain from* Arabidopsis thaliana* PLD*β* (AtPLD*β*) harbors 3 Ca^2+^ binding sites with different affinities within its CBRs (Kd_1_ = 0.8 *μ*M, Kd_2_ = Kd_3_ = 24 *μ*M) [35]. Thus, it has been hypothesized that this dissimilar affinity for Ca^2+^ came from the lack of one acidic residue within the CBR1 of the C2 domain of the AtPLD*α*, due to the substitution of D by Q residue, which could explain the millimolar concentration range of Ca^2+^ required for optimal activity of AtPLD*α* in vitro [35]. However, a recent study on the C2 domain from tomato PLD*α* tends to disclaim these properties, demonstrating the existence of two high-affinity binding sites for Ca^2+^-binding sites (Kd = 59.73 *μ*M) [36].

Interestingly, while the C2 domain of the AtPLD*α* has been extensively investigated, to our knowledge, only one report on the recombinant expression of the whole enzyme has been performed, so far [37]. Although the resulting enzyme was found to be active, it is worth noting that it was expressed in the prokaryotic expression system* E. coli*, which does not allow several eukaryotic and plants-like posttranslational modifications, such as glycosylation. Therefore, here we report the functional expression of the recombinant AtPLD*α* in the eukaryotic expression system* P. pastoris. *The N-terminal amino acid sequence was subsequently determined and was found to lack the first 35 residues belonging to the C2 domain, as found in other recombinant or plant purified PLDs. Thus, to investigate the impact of such a cleavage on the functionality of plant C2 domains, we subsequently expressed the mature-like form of the C2 domain of the AtPLD*α* along with its equivalent C2 domain of the AtPLD*β*, for the sake of comparison. Interestingly, both domains were found to be functional and to be able to bind PG in a Ca^2+^ independent manner while PA and PS binding were found to be enhanced in the presence of Ca^2+^ ions.

## 2. Materials and Methods

### 2.1. Reagents and Materials

1-Palmitoyl-2-oleoyl-*sn*-glycero-3-phosphocholine (POPC), 1-palmitoyl-2-oleoyl-*sn*-glycero-3-phosphoethanolamine (POPE), 1-palmitoyl-2-oleoyl-*sn*-glycero-3-phospho-(1′-rac-glycerol) (POPG), 1-palmitoyl-2-oleoyl-*sn*-glycero-3-phospho-L-serine (POPS), and 1-palmitoyl-2-oleoyl-*sn*-glycero-3-phosphate (POPA) were obtained from Avanti Polar Lipids (Alabaster, Alabama, USA). Protino® Ni-TED and NucleoBond® Xtra Midi packed columns were purchased from Macherey-Nagel GmbH & Co. KG (Germany). Choline oxidase (from* Arthrobacter globiformis*), horseradish peroxidase (type VI), triolein, and monoclonal anti-poly-Histidine coupled to peroxidase antibody were obtained from Sigma-Aldrich-Fluka Chemie (Saint-Quentin-Fallavier, France). Complete protease inhibitors cocktail tablets were purchased from Roche Diagnostic (Mannheim, Germany). Taq polymerase from* Thermus aquaticus* and Zymolyase 20T were purchased from Euromedex (Souffelweyersheim, France). KOD DNA polymerase from* Thermococcus kodakaraensis* and pET28b(+) plasmid were obtained from Novagen (MerckKGaA, Darmstadt, Germany). QIAquick PCR Purification Kit was provided by Qiagen (Courtaboeuf, France). BL21-Codon Plus- (DE3-) RIPL strain was obtained from Agilent Technologies (Courtaboeuf, France). One Shot TOP10 strain and pGAPZB were purchased from Fisher Scientific (Illkirch, France). Restriction enzymes were obtained from either Jena Bioscience GmbH (Loebstedter, Germany) or New England Biolabs (Evry, France).

All solutions were prepared using Milli-Q grade water (Millipore, 18.2 MΩ·cm resistivity).

The* Arabidopsis thaliana* full-length cDNA encoding PLD*α*1 was a gift from Dr. Wang [35] and the* Arabidopsis thaliana* full-length cDNA encoding PLD*β*1 (pdx82318) was provided by the plant genome project at the RIKEN Genomic Sciences Center (Japan).

### 2.2. pGAPZB Modification and Cloning of AtPLD*α*

To bring closer together the GAP promoter and the coding sequence, pGAPZB was modified by digestion using EcoRI and KpnI. After purification of the plasmid, cohesive 5′ and 3′ ends were blunted using T4 DNA polymerase, leading to the reconstruction of the* EcoRI* site along with the destruction of the* KpnI* site, and further joined using T4 DNA ligase. Finally, the vector was amplified in* E. coli* TOP10 strain, purified, and verified by sequencing (Eurofins MWG, Ebersberg, Germany). For subsequent cloning, cDNA sequences coding for the wild type AtPLD*α* (AtPLD*α*-WT) and the poly-histidine tagged (6xHis-Tag) AtPLD*α* (AtPLD*α*-6xHis) were amplified by PCR, using the same forward primer (TCAGTCGACAAAAGA**ATG**GCGCAGCATCTGTTGC), carrying the starting codon and harboring the* SalI* restriction site. Both reverse primers were designed with the* NotI* restriction site in the presence of a stop codon for AtPLD*α*-WT (TGCTGCGGCCGC **TTA**GGTTGTAAGGATTGGAG) or in the absence for AtPLD*α*-6xHis (TGCTGCGGCCGCGGTTGTAAGGATTGGAGG). Subsequently, purified PCR products and modified pGAPZB were, respectively, digested with SalI-NotI and XhoI-NotI and SalI and XhoI harboring compatible cohesive ends and then ligated using T4 DNA ligase. Finally, constructs were amplified, purified, and verified by sequencing as described above.

### 2.3. *Pichia pastoris* Transformation and Cell Lysis

*Pichia pastoris *(X-33 strain) transformation was carried out by electroporation using a Bio-Rad Micropulser, as previously described [38], to obtain recombinant clones transformed by the pGAPZB vector alone or by the vector containing either the AtPLD*α*-WT or the AtPLD*α*-6xHis. Proper genomic insertion of the vector containing either the AtPLD*α*-WT or the AtPLD*α*-6xHis was checked by PCR using primers located on the* GAP* promotor and on the* pld*. Alternatively, proper genomic insertion of the pGAPZB vector alone was checked by PCR using primers located on the* GAP* promotor and the* AOX1* terminator. To follow up the proper expression of the AtPLD*α*-WT or the AtPLD*α*-6xHis, PLD activities were monitored directly from crude protein extracts. To do so, PCR-positives clones, transformed by the pGAPZB vector alone or by the vector containing either the AtPLD*α*-WT or the AtPLD*α*-6xHis, were grown on YPD medium for 4 days at 30°C and harvested by centrifugation at 5,000 ×g. Subsequently, cells were resuspended in a buffer containing 50 mM Tris-HCl, pH 8.0, 150 mM NaCl, and 200 mM sucrose and supplemented with EDTA-free protease inhibitor cocktail tablets (Roche Diagnostics) and 50 *μ*g/mL Zymolyase. After 1 hour of incubation at 30°C, cells were lysed using a Mini-Beadbeater (Biospec), insoluble material from the crude extract was pelleted by centrifugation at 10,000 ×g, and the soluble fraction was tested for PLD activity.

### 2.4. PLD Assay and Protein Determination

PLD activity was assayed spectrophotometrically in microtitration plates as previously described [38]. The amount of free choline generated from the PLD-catalyzed hydrolysis of POPC was quantified colorimetrically, after oxidation to betaine by choline oxidase with simultaneous production of hydrogen peroxide, which allows oxidative coupling of 4-aminoantipyrine and sodium 2-hydroxy-3,5-dichlorobenzene sulfonate by peroxidase to give a maximal absorbance at 500 nm. The substrate solution was prepared by dispersing 1 mM of POPC in an equimolar mixture (3.1 mM) of SDS and Triton X-100. The mixed micelle solution was vortexed for 30 s, sonicated for 10 min using a bath-type sonicator (Deltasonic type O11C, Fécamp, France), and vortexed again for 30 s. The assay mixture (150 *μ*L final volume) was composed of 266 *μ*M POPC, 0.85 mM SDS, 0.85 mM Triton X-100, 50 mM Tris-HCl, pH 8.0, 5.1 mM 4-aminoantipyrine, 27 mM sodium 2-hydroxy-3,5-dichlorobenzene sulfonate, 0.5 U choline oxidase, and 0.5 U peroxidase, the protein extract to be tested, and the reaction was initiated by adding 20 mM CaCl_2_. The PLD-generated choline was continuously quantified by recording the absorbance at 500 nm, based on a standard curve obtained with pure choline. Control assays were performed simultaneously in the absence of PLD. One unit of PLD activity was defined as the amount of enzyme releasing 1 *μ*mol of choline per minute under the experimental conditions specified above. Data are expressed as means ± SD. Statistical significance was determined by Student's unpaired *t*-test (two-tailed). Samples were considered to be significantly different for *P* < 0.05 (*∗*), *P* < 0.01 (*∗∗*), and *P* < 0.001 (*∗∗∗*). The proteins concentration was routinely determined using Bradford's procedure [39] with Bio-Rad Dye Reagent. Bovine serum albumin was used as the reference protein.

### 2.5. Purification of Recombinant AtPLD*α*-WT

For large scale expression of AtPLD*α*-WT, 50 mL of an overnight* P. pastoris* transformant cell culture was inoculated in 1 L of YPD medium and grown at 30°C with orbital shaking (250 rpm) for 6 days. Cells were then harvested by centrifugation at 5,000 ×g and resuspended as described above. Cells were then disrupted by probe sonication and insoluble material was removed by centrifugation at 10,000 ×g for 20 min at 4°C. The soluble fraction was subsequently dialyzed overnight against 30 mM Pipes buffer, pH 6.2, and 50 mM CaCl_2_ and applied onto an octyl-Sepharose CL-4B column (2.5 × 20 cm) equilibrated in the same Pipes buffer. The column was then washed with 10 column volumes of the same buffer followed by a second wash with a buffer containing 10 mM Pipes, pH 6.2, and 30 mM CaCl_2_. The elution of the proteins bound to the column was finally performed with a buffer composed of 10 mM Pipes, pH 6.2, and 0.1 mM EDTA and all purification fractions were assayed for PLD activity. Active fractions were pooled, concentrated by ultrafiltration, and analyzed by SDS-PAGE.

### 2.6. N-Terminal Sequence Analysis

For N-terminal sequencing, eluted proteins from octyl-Sepharose column were analyzed by SDS-PAGE and subsequently electroblotted onto a PVDF membrane for 1 h at 100 V in 90 mM Tris base, 90 mM Boric acid, 0.1% SDS, and 20% (v/v) ethanol transfer buffer using a Trans-Blot apparatus (Bio-Rad). Protein band corresponding to AtPLD*α* was excised and submitted for Edman degradation using an automatic sequencer model Procise 494A from Applied Biosystems.

### 2.7. Cloning and Expression of the 6xHis-Tagged C2*α* and C2*β* Domains

cDNA coding sequences of AtPLD*α* and AtPLD*β* were amplified by PCR with KOD polymerase using forward primers harboring a* NcoI *restriction site. An extra methionine was added to the C2*α* sequence (TGCACC **ATG** GCAAATGTAGAAGAGACG) while the second codon of C2*β* was changed to insert the new restriction site, leading to the substitution of F residue by V residue (TGCACC **ATG** GTTGGAAGATTGCCAGG). Reverse primers for the amplification of the C2*α* domain (TGCTGCGGCCGCCTCAACATGGAAATATTGAAGC) and the C2*β* domain (TGCTGCGGCCGCCATAGGAGTATACTGAATCG) were designed by inserting a* NotI *restriction site in the absence of stop codon. The amplified fragments were subsequently cloned in the Novagen expression vector pET-28b(+) between* NcoI* and* NotI* sites, flushed with a 3′ 6xHis-Tag coding sequence. Constructs were then amplified, purified, and checked by performing DNA sequencing, as described above. All transformations were carried out by electroporation in BL21-CodonPlus® (DE3)-RIPL competent cells, carrying the pRIPL vector. Resulting transformants were grown on LB plates at 37°C, in the presence of kanamycin and chloramphenicol. For the recombinant expression of C2 domains, 2 liters of LB broth was inoculated with 25 mL of an overnight culture, supplemented with appropriate antibiotics. Cells were then grown at 37°C until OD_600 nm_ reached ~0.8 and induced overnight by adding 1 mM isopropyl-1-thio-*β*-galactopyranoside, at 20°C. To evaluate the expression level of both C2 domains, the total proteins extracted from an equivalent of 100 *μ*L of culture at an OD_600 nm_ of 1 was loaded on SDS-PAGE. Cells were finally harvested by centrifugation at 5,000 ×g for 20 min at 4°C and pellets were stored at −20°C, until use.

### 2.8. Purification of C2 Domains

Cell pellets were resuspended in a buffer containing 50 mM NaH_2_PO_4_, pH 8, 300 mM NaCl, and 5 mM reduced glutathione (GSH). Cells were then harvested by probe sonication for 3 × 3 minutes on ice and centrifuged at 5,000 ×g for 30 min at 4°C. As C2 domains were expressed in insoluble inclusion bodies, pellets were collected and washed 3 times with phosphate buffer. C2 domains were then solubilized using urea-containing buffer, composed of 50 mM NaH_2_PO_4_, pH 8, 300 mM NaCl, 1 mM GSH, and 8 M urea. Supernatants were collected by centrifugation at 5,000 ×g for 20 min at 20°C and immediately applied onto a Ni-TED column for purification, using manufacturer's instructions. Briefly, columns were washed with the same buffer followed by the elution of C2 domains bound to the column with a buffer composed of 50 mM NaH_2_PO_4_, pH 8, 300 mM NaCl, 5 mM GSH, 8 M urea, and 250 mM of imidazole. Finally, the eluted fractions were applied onto PD-10 desalting columns, exchanging the elution buffer against a buffer containing 50 mM Tris/HCl, pH 8, 5 mM GSH, and 8 M urea, and C2 domains' purity was evaluated on 15% SDS-PAGE (20 *μ*g of proteins per lane).

### 2.9. Refolding and Intrinsic Fluorescence Analysis of C2 Domains

Small scale refolding trials were carried out by diluting the denatured C2 domains into the refolding buffer, composed of 50 mM Tris/HCl, pH 8, and 5 mM GSH, and incubated for 1 hour at 20°C. The final concentration of both domains was set at 1 *μ*M, leading to a final concentration of urea ranging from 50 mM to 75 mM. For the sake of comparison, both domains were also diluted into the denaturing buffer, corresponding to the refolding buffer but supplemented with 8 M urea. The refolding process was followed by examining the intrinsic fluorescence of both domains, in the absence or in the presence of 8 M urea. Fluorescence experiments were carried out on a Hitachi F-4500 spectrofluorometer in a 1.5 mL quartz cuvette at 20°C. Intrinsic fluorescence spectra were recorded by exciting C2 domains at 280 nm and fluorescence emission wavelengths were scanned from 300 to 500 nm, maintaining both excitation and emission slits widths set at 10 nm. Fluorescence spectra were also recorded in the absence of C2 domains for background corrections due to buffer's Raman scattering. Large scale refolding of C2 domains was carried out as described above, followed by three dialysis steps against the refolding buffer, filtered through a 0.22 *μ*m filter, aliquoted, and stored at −20°C.

### 2.10. Phospholipid Dot-Blot Assay

Lipids (20 nmol) dissolved in CHCl_3_ were spotted onto a PVDF membrane and let to evaporate under fume hood. Similarly, 6xHis-Tagged C2*α* or C2*β* were also spotted onto a PVDF membrane as a control of the revelation by anti-6xHis antibodies. The membrane was blocked in Tris-buffered saline (TBS) supplemented with fatty acid-free BSA (TBS/BSA) at room temperature for 1 hour and subsequently incubated with refolded C2*α*, refolded C2*β*, or the refolding buffer alone, diluted in TBS/BSA at 4°C overnight, in the presence of either 1 mM of Ca^2+^ or 1 mM of EDTA. After washing three times with TBS/BSA, the membrane was incubated one hour in the presence of TBS/BSA supplemented with 0.2% of Tween 20. An anti-6xHis mouse monoclonal antibody coupled to peroxidase was subsequently added and incubated at room temperature for 1 hour. Finally, membranes were washed three times with the same buffer and proteins bound to lipids were revealed using a solution composed of 3,3′-diaminobenzidine tetrahydrochloride and 3% H_2_O_2_.

### 2.11. Förster Resonance Energy Transfer

Relative binding of the C2*α* domain and the C2*β* domain to phospholipids vesicles was carried out by using the protein to dansyl-PE Förster Resonance Energy Transfer (FRET). To do so, phospholipid liposomes were made by the extrusion method, passing seven times the solution through a 100 nm filter membrane, using a mini extruder (Avanti Polar Lipids). Typical liposomes were composed of the desired phospholipid supplemented with 5% of dansyl-PE. Average hydrodynamic diameters of liposomes, in the absence or in the presence of Ca^2+^, were checked by performing dynamic light scattering experiments in a 1 mL cuvette on a Zetasizer Nano ZS (Malvern Instruments Ltd., United Kingdom). Relative bindings were examined by mixing 20 *μ*M of liposomes with 1 *μ*M of C2 domain or the buffer alone in a 50 mM HEPES buffer, pH 8, as previously described [36]. All fluorescence experiments were recorded at 30°C using a Tecan Infinite M200 (Salzburg, Austria) microplate fluorescence reader at excitation and emission wavelengths set at 280 nm and 510 nm, respectively, with excitation and emission slits widths set at 10 nm and 20 nm, respectively. Relative FRET was calculated as (*I* − *I*_0_)/*I*_0_, where *I* and *I*_0_ correspond to the fluorescence intensity of the C2 domain and the buffer alone, at 510 nm. Effect of Ca^2+^ ions was carried out by adding small proportion of CaCl_2_ and incubated 10 minutes at 20°C.

### 2.12. Secondary Structures Predictions and Molecular Modeling

Predictions of secondary structures of C2 domains were carried out with the Psipred server [40]. Molecular modeling of C2 domains was carried out using the I-tasser server [41] and Ca^2+^ ions were manually added, based on structural alignments of C2*α* and C2*β* against several C2 domains with known 3D structures, followed by energy minimization using the Chimera software. These C2 domains are identified in the current study with their Protein Data Bank (PDB) entry and correspond to cPLA_2_ (1RLW), perforin (3W57), Munc13-1 (3KWU), Car4 (5A50 and 4V29), Car4 (5A52), PKC*α* (1DSY), PKC*β* (1A25), rabphilin-3A (2K3H and 2CM5), Synaptotagmin-1 C2A (1BYN), and C2B (1UOV), SV2A (4V11), and Synaptotagmin-7 C2B (3N5A). Membrane interacting residues of these domains were extracted from the OPM database [42] (Orientation of Proteins in Membranes) while C2*α* and C2*β* docking to membranes was carried out using the PPM server (Peripheral Proteins in Membranes) [42].

## 3. Results and Discussion

### 3.1. Yeast Expression of the Recombinant AtPLD*α*

Based on the previous report on the recombinant expression of the* Vigna unguiculata* PLD*α* (VuPLD*α*) [38], we cloned the full-length cDNA encoding the AtPLD*α* into the pGAPZB vector, in the absence (AtPLD*α*-WT) or in the presence of a carboxyterminal polyhistidine tag (AtPLD*α*-6xHis), for the constitutive expression in the yeast* Pichia pastoris*. In contrast to the prokaryotic* E. coli* expression system,* P. pastoris* is a eukaryotic organism that appears very effective for the recombinant expression of eukaryotic proteins [43], allowing various posttranslational modifications such as glycosylation.

**Figure 1:**
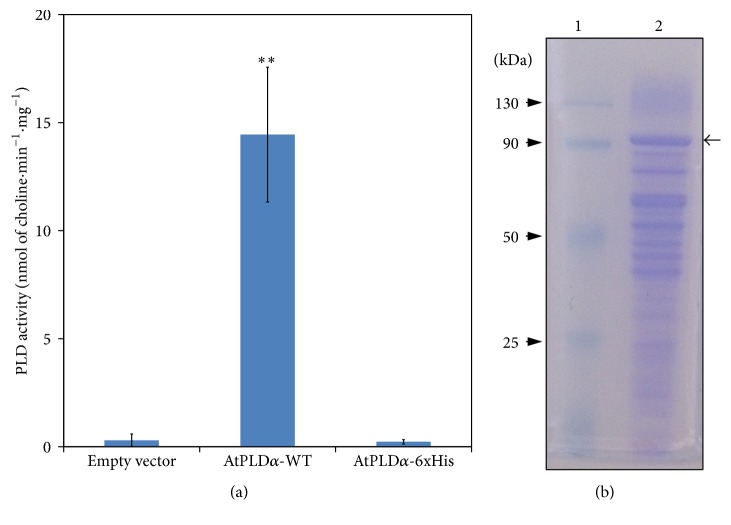
(a) Recombinant AtPLD*α* activity in crude protein extracts of* Pichia pastoris *transformed with the empty vector or expressing either the recombinant AtPLD*α*-WT or the AtPLD*α*-6xHis, after 4 days of culture, and using POPC (0.26 mM, final concentration) as the substrate. Values presented are the means ± SD obtained from four independent PCR-positive clones. ^*∗∗*^ *P* < 0.01 (versus blank). (b) SDS-PAGE (10% acrylamide) analysis of the recombinant AtPLD*α*-WT purified using the purification procedure with octyl-Sepharose CL-4B column (2.5 × 20 cm) (see Materials and Methods). Lane 1: molecular mass marker, lane 2: eluted fraction containing the recombinant AtPLD*α*-WT. The gel was stained with Coomassie Brilliant Blue R-250 to reveal the proteins. The arrow on the right indicates the position of the AtPLD*α*-WT.

Thus,* P. pastoris* (X-33 strain) transformation yielded several colonies harboring a correct genomic recombination, as confirmed by PCR, with the pGAPZB containing the coding sequence of either the AtPLD*α*-WT or the AtPLD*α*-6xHis or with the empty vector, serving as the negative expression control. To investigate the functional expression of both recombinant AtPLDs, PCR-positives clones were grown for several days and further assayed for PLD activity. Therefore, as shown in Figure 1(a), crude extracts of* P. pastoris* transformed with the AtPLD*α*-WT exhibited a higher PLD activity, in comparison to the negative control. Besides the time course expression of the AtPLD*α*-WT showed an increasing specific PLD activity as function of culture time and reaching a maximum at 7 days of culture (data not shown). Interestingly, this result contrast with the previously reported VuPLD*α* expression pattern that showed a rapid increase of PLD activity with a maximum reached after 3 days of culture [38]. Nevertheless, this result indicates a functional expression of the AtPLD*α*-WT and suggests that* P. pastoris* is a well-adapted expression system for the recombinant production of plants PLDs.

It is worth noting that, under the same experimental conditions, no PLD activity could be observed in crude extracts of* P. pastoris* transformed with the sequence coding for AtPLD*α*-6xHis (Figure 1(a)), thus suggesting either a misfolding of the protein due to the 6xHis-tag or a wrong genomic insertion of the cDNA. Interestingly, these results are in line with the previous report of Schäffner et al. [44] who showed that, using the recombinant BoPLD*α*1 and BoPLD*α*2, the addition of an amino- or carboxyterminal tag yielded inactive enzymes.

### 3.2. Partial Purification and N-Terminal Amino Acid Sequencing of Recombinant AtPLD*α*

**Figure 2:**
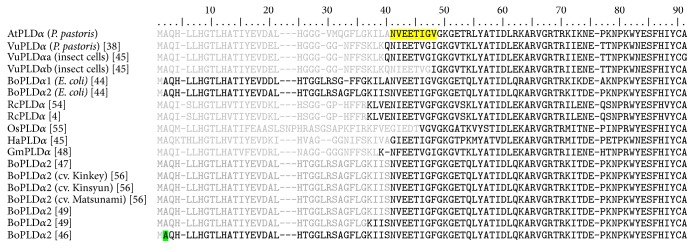
Protein sequence alignment of the N-terminal region of purified *α*-type PLDs reported so far. Grey-colored residues represent predicted sequences deduced from cDNAs and absents in mature PLD forms (black-colored residues). Residues highlighted in yellow come from the Edman degradation sequencing obtained in this study while the posttranslational acetylated residue is highlighted in green.

To further characterize the recombinant AtPLD*α*-WT, we used the property of plant PLDs to strongly bind hydrophobic supports such as octyl-Sepharose in the presence of calcium as previously reported [38, 44–50]. The PLD activity was eluted by chelating the Ca^2+^ ions with EDTA. Partially purified recombinant AtPLD*α*-WT was obtained, as attested by a major protein band observed in the SDS-PAGE analysis (Figure 1(b), arrow), corresponding to a molecular mass, as expected, of around 90 kDa. However, despite a harshly wash of the octyl-Sepharose column during the purification process, an important proportion of contaminant proteins were found in the obtained AtPLD*α*-WT containing elution fractions. Concerning the analysis of the AtPLD*α*-WT (Figure 1(b), arrow), the protein band was subject to Edman degradation and the obtained N-terminal sequence was found to be NVEETIGV. This N-terminal sequence analysis is a clear evidence for the identification of the AtPLD*α*-WT, since BLAST analysis against* Pichia*'s genome did not return results, while this sequence perfectly matches with the AtPLD*α*-WT. As shown in Figure 2, this amino acid sequence corresponds to positions 36 to 43 of the AtPLD*α*-WT and was found to be very similar to the previously obtained N-terminal sequence of the recombinant VuPLD*α* expressed in* P. pastoris* [38].

Taking into account the observed N-terminal cleavage and starting from residue 36, the resulting AtPLD*α*-WT is expected to have a molecular mass of 88.1 kDa (775 amino acid residues), thus contrasting with the observed molecular mass on SDS-PAGE (see Figure 1(b)). Nonetheless, the AtPLD*α*-WT contains one potential N-glycosylation site that may explain the observed difference in the molecular mass. Indeed, since the addition of single N-linked glycans adds ~2.5 kDa [51], the AtPLD*α*-WT is expected to reach an apparent molecular mass of around 90.6 kDa, corresponding thus to the one estimated from SDS-PAGE analysis (see Figure 1(b)).

The* in vivo* glycosylation of *α*-type PLDs has been demonstrated for the* Glycine max* PLD*α* (GmPLD*α*) [48] that reacts with lectins but also with antibodies directed against complex Asn-linked glycans [48]. Besides, a similar glycosylation pattern was found for the* Helianthus annuus* PLD*α* (HaPLD*α*) and the* Brassica oleracea *PLD*α* (BoPLD*α*) (Abousalham et al., unpublished data), purified as previously described [45, 47]. The glycosylation character of these enzymes was established by performing Western blotting, either by reacting HaPLD*α* and BoPLD*α* with specific lectins, such as concanavalin A, wheat germ agglutinin, or* Lens culinaris* agglutinin, or using anti-glycan polyclonal antibodies. The later strategy used fractionated horseradish peroxidase antibodies, purified on an affinity column of honeybee venom phospholipase A_2_, to produce serum fractions that are specific for the *α*(1,3)-fucose epitopes [52]. In plants, the *α*(1,3) linked fucose is commonly found in complex Asn-linked glycans and paucimannosidic Asn-linked glycans [53] and is added in the Golgi apparatus.

Besides,* P. pastoris* is able to recognize the consensus sequence Asn-X-Ser/Thr (where X is any amino acid except proline), which constitute the N-glycosylation sites in yeast but also in mammals and in plants [53], explaining why the recombinant VuPLD*α*, expressed either in* P. pastoris *[38] or in insect cells [45], was also found to be glycosylated.

Interestingly, a significant number of studies have reported this kind of cleavage on *α*-type PLDs purified from plant tissues. Indeed, this phenomenon has been first reported by Wang et al. [4, 54] for the RcPLD*α* and subsequently described for the PLD*α* from* Oryza sativa* (OsPLD*α*) [55], GmPLD*α* [48], and HaPLD*α* [45] as well as for both BoPLD*α*1 and BoPLD*α*2 [47, 49, 56]. However, in a more recent study, Schöps et al. [46] have reported an N-terminal acetylation of the second residue of the BoPLD*α*2, which may explain why attempts to sequence the N-terminal part of the PLD*α* from* Brassica napus* (BnPLD*α*) [57] and* Arachis hypogaea* (AhPLD*α*) [58] did not gave results. Oppositely, the recombinant expression in* E. coli* of both BoPLD*α*1 and BoPLD*α*2 was found to lead to the production of full-length enzymes [44]. Therefore, authors suggested that the observed cleavage may come from a proteolytic degradation during the purification process. Nevertheless, using the insect cells expression system, El Maarouf et al. [45] also reported the purification of two forms of the recombinant VuPLD*α* with distinct N-terminal cleavage sites (VuPLD*α*a and VuPLD*α*b). Interestingly, both forms were found to be secreted in the culture medium without the addition of a secretion peptide, leading authors to hypothesize that the N-terminal part of *α*-type PLDs may act as a leader peptide.

Although the mechanism of such a cleavage or degradation needs further insights, this phenomenon may come from the action of some proteases towards the C2 domain of the PLD*α*. Indeed, in a previous study, Simões et al. [59] reported the interaction between the PLD*α* from* Cynara cardunculus* and cardosin A, an aspartic proteinase which accumulates in protein storage vacuoles [59]. This specific interaction was found to occur within the C2 domain via both a RGD motif and a KGE motif, present in the cardosin A, and resulted in the proteolysis of the GST N-terminal tagged recombinant C2 domain of the* C. cardunculus* PLD*α* upon dissociation of the complex [59]. Interestingly,* P. pastoris *presents several vacuolar proteases that harbor this RGD or KGE motif which is, in a surprising manner, also found in the CBR1 of the AtPLD*α* C2 domain.

### 3.3. Expression, Purification, and Refolding of N-Terminal C2 Domains from AtPLD*α* and AtPLD*β*

**Figure 3:**
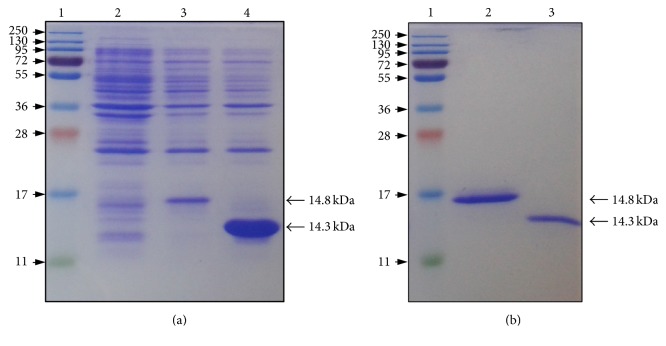
(a) SDS-PAGE analysis of crude protein extracts from* E. coli* cells expressing either the C2*α* domain or the C2*β* domain, in comparison to the empty vector as expression control. Lane 1: molecular mass marker, lane 2: empty vector, lane 3: C2*α* domain, and lane 4: C2*β* domain. (b) SDS-PAGE analysis of affinity purified C2*α* domain and C2*β* domain. Lane 1: molecular mass marker, lane 2: C2*α* domain, and lane 3: C2*β* domain. Protein migrations were performed using 15% SDS-PAGE and stained with Coomassie Brilliant Blue R-250.

Because the exact nature of the removed N-terminal part of *α*-type PLDs remains elusive, we wondered about the impact of such cleavage on the functionality of the C2 domains. To investigate further this concern, we subsequently expressed the mature-like form of the C2 domains from AtPLD*α* (C2*α*) along with its equivalent C2 domain from AtPLD*β* (C2*β*), based on protein sequence alignments, for the sake of comparison. Expression of C2*α* and C2*β* in BL21 cells resulted in a rapid accumulation of proteins in inclusion bodies as shown by SDS-PAGE analysis of* E. coli* crude protein extracts expressing either the C2*α* (Figure 3(a), lane 3) or the C2*β* (Figure 3(a), lane 4). Therefore, C2*α* and C2*β* were solubilized under denaturing conditions using urea and subsequently purified by affinity chromatography using Ni^2+^ affinity resins. SDS-PAGE analysis of purified C2*α* and C2*β* devoid of any detectable contaminants gave a single protein band at a position corresponding to a molecular mass of 14.8 (Figure 3(b), lane 2) and 14.2 kDa (Figure 3(b), lane 3), respectively. Pure C2 domains were subsequently refolded by rapid dilution at low protein concentration in a urea-free buffer and extensively dialyzed to remove urea. However, subsequent concentration attempts by ultra-filtration led to an important protein precipitation, yielding a poor recovery of both C2 domains. The addition of chemical reagents such as arginine or N-lauroylsarcosine were found to drastically enhance the protein solubility; however, both C2 domains were prone to aggregate during subsequent dialysis.

**Figure 4:**
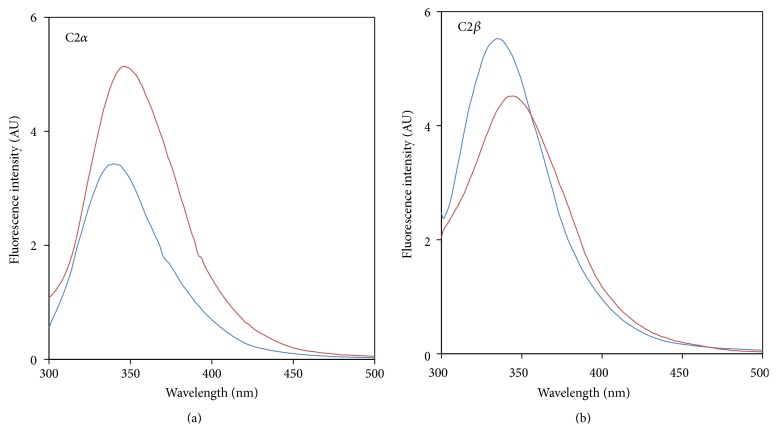
Intrinsic fluorescence spectra (*λ*_ex_ 280 nm) of urea-denatured C2*α* domain (a) and C2*β* domain (b) diluted in a Tris/HCl buffer containing (red curves) or not (blue curves) 8 M urea and after 1 h of incubation at room temperature. The final concentration of both domains was set at 1 *μ*M, leading to a final concentration of urea ranging from 50 mM to 75 mM for blue curves. Fluorescence intensity was expressed in arbitrary units (AU) and bandwidths were set at 10 nm for both excitation and emission.

In these experimental conditions, the maximal concentration that could be obtained was found to be 5 *μ*M (7 *μ*g·mL^−1^) for the C2*α* and 9 *μ*M (13 *μ*g·mL^−1^) for the C2*β*. Despite the low protein concentration obtained, the refolding process of these domains could be followed using their intrinsic fluorescence. Protein unfolding usually leads to the exposure of fluorescent residues (Trp/Tyr), resulting in a modification of the fluorescence signal and a shift of the emission peak. Interestingly, in the presence of 8 M urea, the two denatured C2 domains present a striking difference of their fluorescence emission maxima, corresponding to *λ*_max_ of 349 nm for the C2*α* (Figure 4(a), red curve) and *λ*_max_ of 345 nm for the C2*β* (Figure 4(b), red curve). Upon reduction of urea concentration, both domains present a blue shift of their fluorescence emission maxima, along with a change in their fluorescence intensity. As shown in Figure 4, the C2*α* presents a fluorescence emission maximum at *λ*_max_ of 340 nm with a concomitant intensity decrease (Figure 4(a), blue curve) while the C2*β* presents an emission maximum at *λ*_max_ of 335 nm along with an increase of its fluorescence intensity (Figure 4(b), blue curve). Thus, both domains showed a decreasing emission maximum wavelength of approximately 10 nm upon denaturant removal, which is usually associated with a burial of tyrosine and tryptophan residues in a more hydrophobic environment, indicating a proper refolding.

### 3.4. C2 Domains-Phospholipids Binding Assay

**Figure 5:**
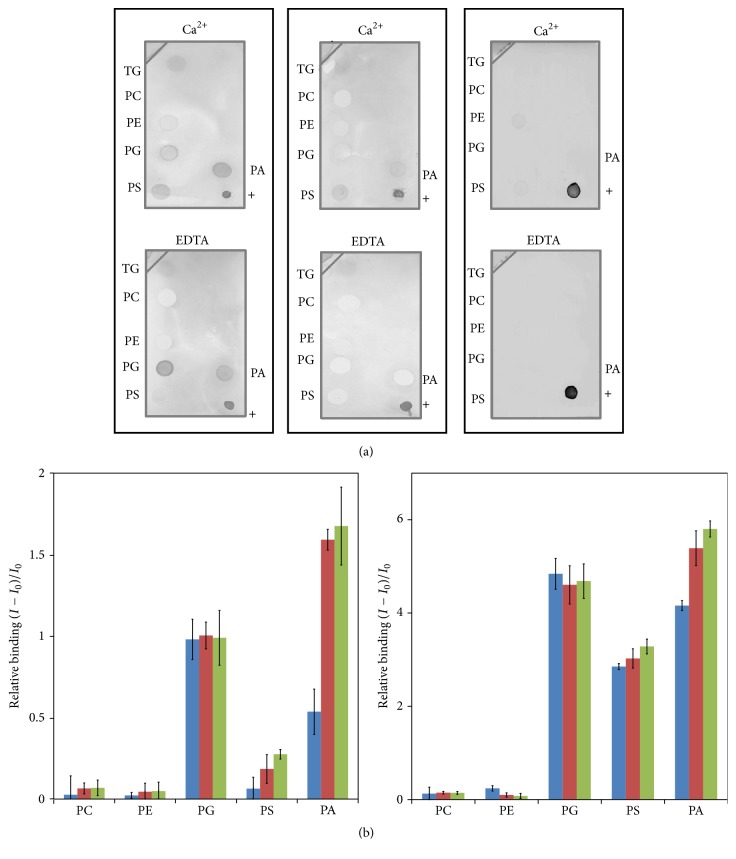
(a) Lipid blot analysis of the interaction of the C2*α* (left panel) and the C2*β* (middle panel) in the presence of 1 mM of Ca^2+^ or 1 mM of EDTA, in comparison to the dialysis buffer serving as the negative control (right panel). Lipids were spotted onto a PVDF membrane and interactions were revealed using an anti-6xHis antibody, as described in the experimental procedures. TG: triglyceride, +: positive control made by spotting 6xHis-C2 domain protein. (b) Relative binding of the C2*α* (left panel) and the C2*β* (right panel) to phospholipids vesicles, using the protein to dansyl-PE FRET. Blue bars: no Ca^2+^ added, red bars: 50 *μ*M of Ca^2+^, and green bars: 200 *μ*M of Ca^2+^. Values presented are the means ± SD obtained from three independent experiments.

To investigate further the impact of the loss of the N-terminal part on the C2 domains properties, the Ca^2+^-dependent or -independent binding of both C2*α* and C2*β* towards phospholipids was examined using the phospholipid dot-blot technique, as indicated in Materials and Methods. As shown in Figure 5(a), both C2 domains clearly bound to anionic phospholipids (PS and PA) in the presence of 1 mM of Ca^2+^. Conversely, the C2*α* bound PG with high affinity in the absence of Ca^2+^ and the affinity decreased upon the addition of Ca^2+^.

These results were further confirmed by FRET experiments, using dansyl-PE labeled liposomes (Figure 5(b)). In these experiments, tryptophan of the C2 domains serves as the donor of fluorescence that is transferred to the dansyl acceptor upon binding to phospholipid vesicles. As shown in Figure 5(b) and as expected, both C2 domains were found to bind anionic phospholipids (PA and PS) in the presence of Ca^2+^, while no affinity could be observed for neutrally charged phospholipids, PC and PE, in either the presence or the absence of Ca^2+^. Interestingly, in the absence of Ca^2+^, both C2 domains showed a strong binding to PG vesicles that was not influenced by the addition of Ca^2+^. Similarly, a slight binding to PS vesicles and a much more pronounced binding to PA vesicles could be observed for both C2 domains, in the absence of Ca^2+^ (Figure 5). It is worth noticing that, in contrast to PG vesicles, the addition of Ca^2+^ was found to enhance binding of both C2 domains to PA and PS (Figure 5). Nevertheless, in comparison to the C2*α*, the C2*β* appeared to require a much less important Ca^2+^ concentration for an efficient binding to PS vesicles.

In contrast to the Ca^2+^-independent phospholipids binding of the C2 domain of tomato PLD*α* [36], these results are in line with the previous report by Zheng et al. [35], who demonstrated that both the recombinant full-length C2*α* of AtPLD*α* and C2*β* ^Δ173–190^ of AtPLD*β* were able to bind PS containing vesicles in a Ca^2+^-dependent fashion, in vitro [35]. Similar to the present study, C2*β* ^Δ173–190^ was found to require much less Ca^2+^ to bind PS vesicles than the C2*α*. This Ca^2+^-dependent binding to PS was thus proposed to be involved in the recruitment of PLDs to the membrane, as demonstrated for the C2 domain of the PKC*α* [60]. Interestingly, the binding to PA and PG vesicles of the C2*α* and the C2*β* presented in this study is also in line with the previous report on the activation of the BoPLD*α* by PA and PG [61] as well as of the HaPLD*α* by PG [20]. This behavior was also demonstrated by Majd et al. [62], who proposed a novel interfacial kinetic model for PLDs, taking into account the activating role of the newly PLD-generated PA. In this model, the PLD activity towards the PC substrate starts with a slow activity phase, which gradually accelerates upon PA accumulation. Interestingly, it is well known that both SDS and Triton X-100 are found to be critical for an optimal PLD activity, in vitro [63]. Also, the addition of these detergents to phospholipids leads to an optimal substrate accessibility through the formation of mixed micelles; we can hypothesize that the negative charge of SDS may mimic PA, thus leading to the activation of *α*-type PLDs.

### 3.5. Secondary Structure Prediction and Molecular Modeling of C2*α* and C2*β*

**Figure 6:**
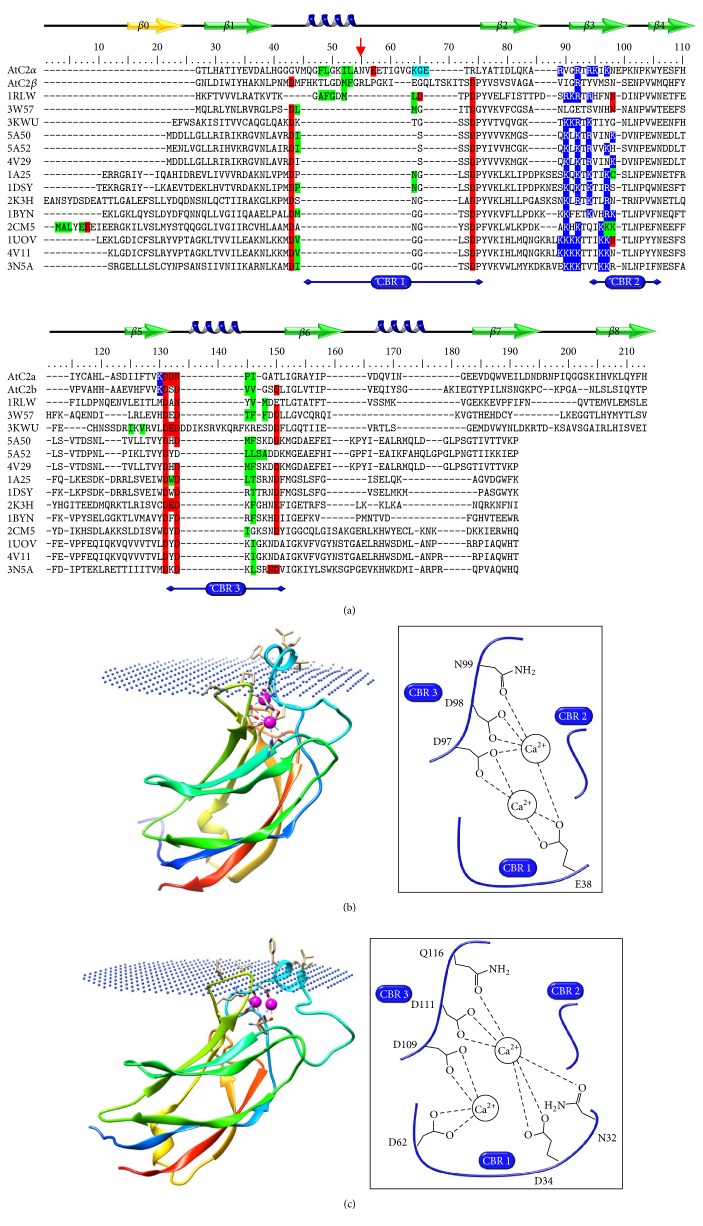
(a) Multiple sequence alignment of C2 domains. Amino acid sequences of C2*α* and C2*β* were aligned with various known C2 domain structures (see Materials and Methods). Residues highlighted in red represent Ca^2+^ binding residues extracted from structures and residues highlighted in green represent membrane binding residues extracted from the OPM database. The proposed *α*-helix between *β*1 and *β*2 strands is indicated in blue. CBR: Ca^2+^ binding region. Strand *β*0, colored in yellow, corresponds to the first *β* strand present in typology I. The observed cleavage on the recombinant AtPLD*α* is indicated by the arrow (red). The PDB entries correspond to cPLA_2_ (1RLW), perforin (3W57), Munc13-1 (3KWU), Car4 (5A50 and 4V29), Car4 (5A52), PKC*α* (1DSY), PKC*β* (1A25), rabphilin-3A (2K3H and 2CM5), Synaptotagmin-1 C2A (1BYN), and C2B (1UOV), SV2A (4V11), and Synaptotagmin-7 C2B (3N5A). Modeled structures of the C2*α* domain (b) and the C2*β* domain (c), using the I-tasser server and docked to membrane (dummy) using the PPM server. Ca^2+^ is colored in pink.

C2*α* and C2*β* are expected to present the 8 conserved *β* strands, as demonstrated by their secondary structure predictions (Figure 6(a)). However, all C2 domains from plant PLDs present, in addition, an extra region between the two predicted *β*1 and *β*2 strands. This singular characteristic relies on an insertion of 15 to 20 amino acid residues that has been previously [35] proposed to fold as an *α*-helix and that agrees with the proposed prediction (Figure 6(a)). Interestingly, the observed cleavage on the recombinant AtPLD*α* appears to occur at the end of the predicted helix, indicated by an arrow in Figure 6(a).

Therefore, to investigate further the property of the predicted *α*-helix, three-dimensional (3D) structures of both the C2*α* and the C2*β* were modeled, based on their secondary structure prediction. As expected, both C2 domains present a *β*-sandwich fold (Figures 6(b) and 6(c)), composed of 8 antiparallel *β* sheets along with the predicted helix. Interestingly, this helix was found to adopt an amphipathic pattern, exhibiting its hydrophobic side outwards from the surface, in a similar manner to the one occurring in the C2 domain of the cPLA_2_ [64], which may explain the observed Ca^2+^-independent membrane binding of the full-length C2 domain from tomato PLD*α* [36]. Besides, in the structure of the C2 domain of the cPLA_2_, the hydrophobic part of the helix appears to interact with the membrane, surrounding the Ca^2+^-binding pocket, and is directly followed by the first Ca^2+^-binding D residue [64]. Interestingly, structural alignments between the two modeled domains and various known C2 domain structures showed a similar structure, allowing us to append Ca^2+^ to CBRs and to predict Ca^2+^-binding residues. Thus, as shown in Figure 6, C2*α* and C2*β* were found to exhibit the conserved D residues of the CBR3 (D97 and D98 for the C2*α* and D109 and D111 for the C2*β*) but, in contrast, presented striking difference in their CBR1. Indeed, while the C2*β* harbors the conserved D residues within its CBR1 (D34 and D62, Figures 6(a) and 6(c)), the C2*α* shows a D to Q substitution (Q49, Figures 6(a) and 6(b)), as previously described [35]. Nevertheless, a novel Ca^2+^-binding (E38) site within the sequence motif “NVEE” could be identified just after the first helix of the C2*α* (Figure 6(a)), similar to the first Ca^2+^-binding D residue of the C2 domain of the cPLA_2_ [64], suggesting that the C2*α* may present a functional CBR1. Interestingly, this Ca^2+^-binding motif is highly conserved among the *α*-type PLDs (see Figure 2) and remains present in the mC2*α* as well as in the matured form of the AtPLD*α*-WT, thus explaining the requirement of a complete chelating of Ca^2+^ during the elution step of the purification process. Besides, this finding is in line with the previous report on the VuPLD*α* produced in insect cells, which showed distinct N-terminal cleavage sites along with different binding properties towards hydrophobic supports in the presence of Ca^2+^. Indeed, the 3-day postinfection purified form, VuPLD*α*a, exhibited an N-terminal sequence similar to the VuPLD*α* expressed and purified in* P. pastoris *and required the total chelating of Ca^2+^ to be eluted from the octyl-Sepharose column [45]. In contrast, the 4-day postinfection purified form, VuPLD*α*b, showed an extra cleavage site of 8 more residues, including the newly predicted Ca^2+^-binding residue, and could be eluted in the presence of only 30 mM of Ca^2+^, suggesting that the CBR1 of the C2 domain was affected [45]. Interestingly, the presence of this Ca^2+^-binding site in the mC2*α* can also explain the observed Ca^2+^-dependent binding to PA and PS, similar to the C2 domain of the PKC*α* [60]. In contrast, the Ca^2+^-independent binding of mC2*α* and mC2*β* to PG suggests the presence of an additional lipid binding site within the C2 domain. This second binding site can correspond to the polybasic motif, previously identified as a Ca^2+^-independent PIP_2_ binding motif [35]. Indeed, as demonstrated on the 3D structure of the C2 domain of the PKC*α* [65], this motif was shown to present several basic residues, within the *β*3 strand (Figure 6(a), 1DSY), which taken together form a highly positively charged patch, thus enabling the binding of anionic phospholipids. Interestingly, this second site may also explain the slight Ca^2+^-independent binding to PS and PA of both domains observed in the current study and as previously reported for PS vesicles [35]. Nevertheless, while the C2*α* exhibits several basic residues within its *β*3 strand (Figure 6(a)), the C2*β* harbors only one R residue, R76, (Figure 6(a)). Therefore, this slight Ca^2+^-independent binding to anionic phospholipids may also be explained by the presence of the K residue (K96 and K108, resp., for the C2*α* and the C2*β*), juxtaposed to the conserved D of the CBR3 of the two domains (Figure 6(a)), as demonstrated by Ananthanarayanan et al. [66], for the PLC-*δ*4. Indeed, this isoform harbors two cationic residues, present within its CBR3, which have been shown to be involved in nonspecific electrostatic interactions with anionic phospholipids [66].

Therefore, both C2 domains (mC2*α* and mC2*β*) may target the membrane through two different binding sites with variable affinities as function of the Ca^2+^ availability and of the lipid composition of membranes. In addition, these two domains may be anchored in the membrane via hydrophobic residues present near the CBR3, as found using the PPM prediction server (Figure 6(a)), even when the amphipathic helix of the CBR1 is removed. Indeed, hydrophobic residues of the CBR3 appear highly conserved in an important number of C2 domains (Figure 6(a)) and are thought to be critical for an efficient membrane binding.

## 4. Conclusion

We report here the functional expression of the recombinant AtPLD*α* in the eukaryotic system* P. pastoris*. Interestingly, while the addition of a C-terminal 6xHis-tag did not give any detectable enzyme, the wild type AtPLD*α* was found to be expressed as an active form, confirming the efficiency of* P. pastoris* for the heterologous production of plant PLDs. In addition, the N-terminal amino acid sequencing of the recombinant AtPLD*α* demonstrated an N-terminal cleavage, at the beginning of the C2 domain, which appears highly similar to the previously purified *α*-type PLDs.

The mature-like form of the C2 domains C2*α* and C2*β* were expressed in* E. coli*, purified, and refolded. Both C2 domains were found to be functional and to be able to bind PG in a Ca^2+^-independent manner while PA and PS bindings were found to be enhanced in the presence of Ca^2+^. Such bindings were found to be in line with the previously activating roles of these phospholipids towards *α*-type PLDs and indicated, in addition, that the matured-like C2 domain of the AtPLD*α* presents all of the conserved Ca^2+^ binding residues. Indeed, molecular modeling of the C2*α* domain 3D structure predicted the presence of a novel Ca^2+^ binding site which may replace the D to Q substitution, within the CBR1. Interestingly, this binding site was found to be highly conserved among *α*-type PLDs and can explain the different binding affinities of the two forms of VuPLD, expressed in insect cells, towards hydrophobic supports in the presence of Ca^2+^.

Taken together, these results suggest that *α*-type PLDs present complete C2 domains that harbor all of the conserved acidic Ca^2+^-binding residues within their CBR1 and CBR3. Besides, our data indicate that these C2 domains are able to bind anionic phospholipids, which is in line with the previous reports on the activation effect of these lipids on the *α*-type PLDs. It can thus be hypothesized that *α*-type PLDs are regulated by their C2 domains through both Ca^2+^ dependent and Ca^2+^-independent binding to anionic lipids.

## References

[1] Selvy P. E., Lavieri R. R., Lindsley C. W., Brown H. A. (2011). Phospholipase D: enzymology, functionality, and chemical modulation. *Chemical Reviews*.

[2] Yang S. F., Freer S., Benson A. A. (1967). Transphosphatidylation by phospholipase D. *Journal of Biological Chemistry*.

[3] Hanahan D. J., Chaikoff I. L. (1947). The phosphorus-containing lipides of the carrot. *The Journal of Biological Chemistry*.

[4] Wang X., Xu L., Zheng L. (1994). Cloning and expression of phosphatidylcholine-hydrolyzing phospholipase D from *Ricinus communis* L.. *The Journal of Biological Chemistry*.

[5] Wang X. (2000). Multiple forms of phospholipase D in plants: the gene family, catalytic and regulatory properties, and cellular functions. *Progress in Lipid Research*.

[6] Li M., Hong Y., Wang X. (2009). Phospholipase D- and phosphatidic acid-mediated signaling in plants. *Biochimica et Biophysica Acta (BBA)—Molecular and Cell Biology of Lipids*.

[7] Dyer J. H., Ryu S. B., Wang X. (1994). Multiple forms of phospholipase D following germination and during leaf development of castor bean. *Plant Physiology*.

[8] Ryu S. B., Zheng L., Wang X. (1996). Changes in phospholipase D experession in soybeans during seed development and germination. *JAOCS, Journal of the American Oil Chemists' Society*.

[9] Hong Y., Devaiah S. P., Bahn S. C. (2009). Phospholipase D*ε* and phosphatidic acid enhance Arabidopsis nitrogen signaling and growth. *Plant Journal*.

[10] Frank W., Munnik T., Kerkmann K., Salamini F., Bartels D. (2000). Water deficit triggers phospholipase D activity in the resurrection plant *Craterostigma plantagineum*. *The Plant Cell*.

[11] Sang Y., Cui D., Wang X. (2001). Phospholipase D and phosphatidic acid-mediated generation of superoxide in Arabidopsis. *Plant Physiology*.

[12] Pinosa F., Buhot N., Kwaaitaal M. (2013). *Arabidopsis* phospholipase Ddelta is involved in basal defense and nonhost resistance to powdery mildew fungi. *Plant Physiology*.

[13] Ryu S. B., Wang X. (1996). Activation of phospholipase D and the possible mechanism of activation in wound-induced lipid hydrolysis in castor bean leaves. *Biochimica et Biophysica Acta—Lipids and Lipid Metabolism*.

[14] Hong Y., Pan X., Welti R., Wang X. (2008). Phospholipase D*α*3 is involved in the hyperosmotic response in *Arabidopsis*. *The Plant Cell*.

[15] Fan L., Zheng S., Wang X. (1997). Antisense suppression of phospholipase D*α* retards abscisic acid- and ethylene-promoted senescence of postharvest arabidopsis leaves. *The Plant Cell*.

[16] Pappan K., Austin-Brown S., Chapman K. D., Wang X. (1998). Substrate selectivities and lipid modulation of plant phospholipase D*α*,-*β*, and -*γ*. *Archives of Biochemistry and Biophysics*.

[17] Moreno-Pérez A. J., Martínez-Force E., Garcés R., Salas J. J. (2010). Phospholipase D*α* from sunflower (*Helianthus annuus*): cloning and functional characterization. *Journal of Plant Physiology*.

[18] Dippe M., Ulbrich-Hofmann R. (2009). Spectrophotometric determination of phosphatidic acid via iron(III) complexation for assaying phospholipase D activity. *Analytical Biochemistry*.

[19] Ella K. M., Meier K. E., Kumar A., Zhang Y., Meier G. P. (1997). Utilization of alcohols by plant and mammalian phospholipase D. *Biochemistry and Molecular Biology International*.

[20] Abdelkafi S., Abousalham A. (2011). The substrate specificities of sunflower and soybean phospholipases D using transphosphatidylation reaction. *Lipids in Health and Disease*.

[21] Rahier R., Noiriel A., Abousalham A. (2016). Development of a direct and continuous phospholipase D assay based on the chelation-enhanced fluorescence property of 8-hydroxyquinoline. *Analytical Chemistry*.

[22] Pappan K., Wang X. (1999). Plant phospholipase D*ε* is an acidic phospholipase active at near- physiological Ca^2+^ concentrations. *Archives of Biochemistry and Biophysics*.

[23] Pleskot R., Potocký M., Pejchar P. (2010). Mutual regulation of plant phospholipase D and the actin cytoskeleton. *The Plant Journal*.

[24] Zhao J., Wang C., Bedair M. (2011). Suppression of phospholipase D*γ*s confers increased aluminum resistance in *Arabidopsis thaliana*. *PLoS ONE*.

[25] Li W., Li M., Zhang W., Welti R., Wang X. (2004). The plasma membrane-bound phospholipase D*δ* enhances freezing tolerance in arabidopsis thaliana. *Nature Biotechnology*.

[26] Wang C., Wang X. (2001). A novel phospholipase D of arabidopsis that is activated by oleic acid and associated with the plasma membrane. *Plant Physiology*.

[27] Nishizuka Y. (1988). The molecular heterogeneity of protein kinase C and its implications for cellular regulation. *Nature*.

[28] Perin M. S., Friedt V. A., Mignery G. A., Jahn R., Südhof T. C. (1990). Phospholipid binding by a synaptic vesicle protein homologous to the regulatory region of protein kinase C. *Nature*.

[29] Clark J. D., Lin L.-L., Kriz R. W. (1991). A novel arachidonic acid-selective cytosolic PLA2 contains a Ca^2+^-dependent translocation domain with homology to PKC and GAP. *Cell*.

[30] Nalefski E. A., Sultzman L. A., Martin D. M. (1994). Delineation of two functionally distinct domains of cytosolic phospholipase A2, a regulatory Ca(2+)-dependent lipid-binding domain and a Ca(2+)-independent catalytic domain. *The Journal of Biological Chemistry*.

[31] Rhee S. G. (2001). Regulation of phosphoinositide-specific phospholipase C. *Annual Review of Biochemistry*.

[32] Nalefski E. A., Falke J. J. (1996). The C2 domain calcium-binding motif: structural and functional diversity. *Protein Science*.

[33] Cho W., Stahelin R. V. (2006). Membrane binding and subcellular targeting of C2 domains. *Biochimica et Biophysica Acta—Molecular and Cell Biology of Lipids*.

[34] Stahelin R. V., Cho W. (2001). Roles of calcium ions in the membrane binding of C2 domains. *The Biochemical Journal*.

[35] Zheng L., Krishnamoorthi R., Zolkiewski M., Wang X. (2000). Distinct Ca^2+^ binding properties of novel C2 domains of plant phospholipase D*α* and *β*. *Journal of Biological Chemistry*.

[36] Tiwari K., Paliyath G. (2011). Cloning, expression and functional characterization of the C2 domain from tomato phospholipase D*α*. *Plant Physiology and Biochemistry*.

[37] Zhao J., Wang X. (2004). Arabidopsis Phospholipase D*ε*1 Interacts with the Heterotrimeric G-protein *α*-Subunit through a Motif Analogous to the DRY Motif in G-protein-coupled Receptors. *Journal of Biological Chemistry*.

[38] Ben Ali Y., Carrière F., Abousalham A. (2007). High-level constitutive expression in *Pichia pastoris* and one-step purification of phospholipase D from cowpea (*Vigna unguiculata* L. Walp). *Protein Expression and Purification*.

[39] Bradford M. M. (1976). A rapid and sensitive method for the quantitation of microgram quantities of protein utilizing the principle of protein-dye binding. *Analytical Biochemistry*.

[40] Buchan D. W. A., Minneci F., Nugent T. C. O., Bryson K., Jones D. T. (2013). Scalable web services for the PSIPRED Protein Analysis Workbench. *Nucleic Acids Research*.

[41] Yang J., Yan R., Roy A., Xu D., Poisson J., Zhang Y. (2015). The I-TASSER suite: protein structure and function prediction. *Nature Methods*.

[42] Lomize M. A., Pogozheva I. D., Joo H., Mosberg H. I., Lomize A. L. (2012). OPM database and PPM web server: resources for positioning of proteins in membranes. *Nucleic Acids Research*.

[43] Daly R., Hearn M. T. W. (2005). Expression of heterologous proteins in *Pichia pastoris*: a useful experimental tool in protein engineering and production. *Journal of Molecular Recognition*.

[44] Schäffner I., Rücknagel K.-P., Mansfeld J., Ulbrich-Hofmann R. (2002). Genomic structure, cloning and expression of two phospholipase D isoenzymes from white cabbage. *European Journal of Lipid Science and Technology*.

[45] El Maarouf H., Carrière F., Rivière M., Abousalham A. (2000). Functional expression in insect cells, one-step, purification and characterization of a recombinant phospholipase D from cowpea (*Vigna unguiculata* L. Walp). *Protein Engineering*.

[46] Schöps R., Schierhorn A., Schäffner I., Mansfeld J., Ulbrich-Hofmann R. (2002). Identification of phospholipase D from cabbage as N-terminally acetylated PLD2. *Journal of Protein Chemistry*.

[47] Abousalham A., Riviere M., Teissere M., Verger R. (1993). Improved purification and biochemical characterization of phospholipase D from cabbage. *Biochimica et Biophysica Acta—General Subjects*.

[48] Abousalham A., Teissere M., Gardies A. M., Verger R., Noat G. (1995). Phospholipase D from soybean (Glycine max L.) suspension-cultured cells: purification, structural and enzymatic properties. *Plant and Cell Physiology*.

[49] Sato H., Watanabe T., Sagane Y., Nakazawa Y., Takano K. (2000). Purification and characterization of phospholipase D from cabbage leaves. *Food Science and Technology Research*.

[50] Lambrecht R., Ulbrich-Hofmann R. (1992). A facile purification procedure of phospholipase D from cabbage and its characterization. *Biological Chemistry Hoppe-Seyler*.

[51] Kornfeld R., Kornfeld S. (1985). Assembly of asparagine-linked oligosaccharides. *Annual Review of Biochemistry*.

[52] Faye L., Gomord V., Fitchettelaine A. C., Chrispeels M. J. (1993). Affinity purification of antibodies specific for asn-linked glycans containing *α*1 → 3 fucose or *β*1 → 2 Xylose. *Analytical Biochemistry*.

[53] Strasser R. (2016). Plant protein glycosylation. *Glycobiology*.

[54] Wang X., Dyer J. H., Zheng L. (1993). Purification and immunological analysis of phospholipase D from castor bean endosperm. *Archives of Biochemistry and Biophysics*.

[55] Ueki J., Morioka S., Komari T., Kumashiro T. (1995). Purification and characterization of phospholipase D (PLD) from rice (*Oryza sativa* L.) and cloning of cDNA for PLD from rice and maize (*Zea mays* L.). *Plant and Cell Physiology*.

[56] Watanabe T., Sato H., Sagane Y., Nakazawa Y., Takano K. (1999). Primary structure of phospholipase D purified from cabbage leaves. *Seibutsu Butsuri Kagaku*.

[57] Novotná Z., Káš J., Daussant J., Sajdok J., Valentová O. (1999). Purification and characterisation of rape seed phospholipase D. *Plant Physiology and Biochemistry*.

[58] Nakazawa Y., Sato H., Uchino M., Takano K. (2006). Purification, characterization and cloning of phospholipase D from peanut seeds. *Protein Journal*.

[59] Simões I., Mueller E.-C., Otto A. (2005). Molecular analysis of the interaction between cardosin A and phospholipase D*α*. Identification of RGD/KGE sequences as binding motifs for C2 domains. *The FEBS Journal*.

[60] Verdaguer N., Corbalan-Garcia S., Ochoa W. F., Fita I., Gómez-Fernández J. C. (1999). Ca^2+^ bridges the C2 membrane‐binding domain of protein kinase C*α* directly to phosphatidylserine. *EMBO Journal*.

[61] Kuppe K., Kerth A., Blume A., Ulbrich-Hofmann R. (2008). Calcium-induced membrane microdomains trigger plant phospholipase D activity. *Chembiochem*.

[62] Majd S., Yusko E. C., Yang J., Sept D., Mayer M. (2013). A model for the interfacial kinetics of phospholipase D activity on long-chain lipids. *Biophysical Journal*.

[63] Abousalham A., Nari J., Teissère M., Ferté N., Noat G., Verger R. (1997). Study of fatty acid specificity of sunflower phospholipase D using detergent/phospholipid micelles. *European Journal of Biochemistry*.

[64] Perisic O., Fong S., Lynch D. E., Bycroft M., Williams R. L. (1998). Crystal structure of a calcium-phospholipid binding domain from cytosolic phospholipase A2. *Journal of Biological Chemistry*.

[65] Guerrero-Valero M., Ferrer-Orta C., Querol-Audí J. (2009). Structural and mechanistic insights into the association of PKC*α*-C2 domain to PtdIns(4,5)P_2_. *Proceedings of the National Academy of Sciences of the United States of America*.

[66] Ananthanarayanan B., Das S., Rhee S. G., Murray D., Cho W. (2002). Membrane targeting of C2 domains of phospholipase C-*δ* isoforms. *The Journal of Biological Chemistry*.

